# Paroxysmal atrial fibrillation and hemochromatosis: a narrative review

**DOI:** 10.1097/MS9.0000000000001605

**Published:** 2023-12-04

**Authors:** Gulzar Ahmed, Sushma Rathi, Harmandeep K. Sidhu, Momil Muzaffar, Muhammad H. Wajid, Komal Kumari, Hajar Fakhor, Nada M. Attia, Koushik Majumder, Vikash Kumar, Usha Tejwaney, Nanik Ram

**Affiliations:** a Dow Medical College; b Dow University of Health Sciences; c Aga Khan University Hospital; d Ziauddin Medical University, Karachi; e King Edward Medical University; f CMH Lahore Medical College, Lahore, Pakistan; g Asselin Hedelin Hospital, Yvetot, France; h Zagazig University, Zagazig, Egypt; i Chittagong Medical College, Chittagong, Bangladesh; j The Brooklyn Hospital Center, New York; k Valley Health System, New Jersey, USA; l Dayanand Medical College and Hospital, Ludhiana, India

**Keywords:** atrial fibrillation, cardiac arrhythmia, hemochromatosis, iron accumulation

## Abstract

Paroxysmal atrial fibrillation (PAF) and hemochromatosis have a complex relationship. This review explores its mechanisms, prevalence, correlations, and clinical manifestations. Hereditary hemochromatosis (HH) involves iron overload due to HFE protein mutations, while atrial fibrillation (AF) is characterized by irregular heart rhythms. Iron overload in hemochromatosis can promote cardiac arrhythmias. AF is prevalent in developed countries and may be linked to cryptogenic strokes. Genetic variations and demographic factors influence the occurrence of both conditions. HH affects multiple organ systems, including the heart, while AF causes palpitations and reduced exercise tolerance. Diagnosis involves iron markers, genotypic testing, and electrocardiogram (ECG) findings. Treatment strategies focus on reducing iron levels in hemochromatosis and managing AF through antithrombotic therapy and rhythm control. Untreated hemochromatosis carries a higher risk of complications, and PAF is associated with increased cardiovascular-related mortality. For better understanding of the mechanisms and to improve management, additional studies are required. Tailored approaches and combined treatments may enhance patient outcomes.

## Introduction

Highlights

- The association between paroxysmal atrial fibrillation (PAF) and hemochromatosis is explored, focusing on the underlying mechanisms of each condition. Hemochromatosis involves mutations in the HFE protein, leading to iron overload, while atrial fibrillation (AF) is characterized by triggers and substrates that initiate and maintain the arrhythmia.
- Epidemiological studies reveal the prevalence of AF in developed countries, and genetic variations associated with iron status biomarkers are linked to an increased risk of AF. Demographic factors influence the occurrence of both AF and hemochromatosis, establishing correlations between these conditions.
- Hemochromatosis can affect multiple organ systems, including the heart, while PAF commonly presents with palpitations and reduced exercise tolerance. Treatment strategies involve reducing iron levels through phlebotomy for hemochromatosis and employing antithrombotic therapy, rate control, and rhythm control for PAF. Early diagnosis and regular treatment are crucial for better prognosis and outcomes in both conditions.

The connection between paroxysmal atrial fibrillation (PAF) and hemochromatosis can be explained by the fundamental processes of each condition. By comprehending their mechanisms, we can gain a better understanding of how these two conditions are related. PAF is a distinct form of atrial fibrillation (AF) where patients experienced intermittent episodes or paroxysms of irregular and rapid heart rhythm^1^. The pathophysiological mechanisms of PAF are complex and not fully elucidated; however, there is a substantial body of evidence suggesting that ectopic atrial premature beats commonly serve as triggers for PAF episodes^2^. Hemochromatosis is a condition characterized by an excessive accumulation of iron throughout the body, which is primarily caused by a deficiency of hepcidin. Hepcidin plays a crucial role in regulating iron transport into the bloodstream. In hemochromatosis, there is a genetic basis for this condition, often resulting from a deficiency in hepcidin–ferroportin binding, which can be attributed to decreased production or activity of hepcidin. From the underlying pathophysiology of both these conditions, we understand that accumulation of iron and its effect on the consistency of blood as well as its detrimental effects on the heart muscles serve as a linking point between the prognosis of hemochromatosis and AF^3^. The prevalence of AF in the general population of developed Europe and North American countries has been estimated to range between 0.5% and 1% in the general population, according to important epidemiological studies conducted in the late 20th and early 21st centuries^4,5^. PAF has gained an increasing attention due to its potential association with cryptogenic strokes. Understanding the cause-and-effect relationship between temporary episodes of AF after a stroke is crucial for guiding treatment decisions. Studies have demonstrated that using anticoagulants is more effective than antiplatelet therapy in managing ischemic strokes linked to AF^6,7^. The utilization of anticoagulants can also serve as an indication that individuals with hemochromatosis, a genetic condition characterized by excess iron absorption, may face an elevated risk of developing AF due to the increased viscosity of their blood^7^. It is worth noting that hemochromatosis is the most prevalent genetic disease in North America and is commonly found in people of northern European descent. Notably, there is a strong association with Celtic populations^8^. It is important to note that the Celtic empire extended beyond traditional Celtic countries, encompassing regions such as northern Portugal and Turkey. Hemochromatosis is most common in European countries like Ireland, France, and Denmark^9^. Looking at this information, we can see that both hemochromatosis and the other disease mentioned are most common in developed countries in Europe and North America^9^. Hemochromatosis can cause a wide range of health issues because it affects many parts of the body. Aside from liver problems that can lead to cirrhosis, having too much iron from hemochromatosis can also harm the heart, pancreas, skin, pituitary gland, reproductive organs, and joints^10^. Many studies have identified the most common signs of hemochromatosis, which include cirrhosis, feeling very tired, skin turning darker, lower interest in sex, shrinkage of testicles, diabetes, stomach pain, and joint pains^11,11^.

## PAF and hemochromatosis

AF is a prevalent cardiac arrhythmia characterized by an irregular and often rapid heart rhythm. It occurs when the electrical signals in the atria become disorganized, resulting in chaotic atrial contractions. In a normal heart rhythm, the atria contract in a coordinated manner due to electrical signals originating from the sinoatrial (SA) node. However, in AF, abnormal signals arise from various locations within the atria, leading to rapid and irregular firing of electrical impulses. As a consequence, the atria quiver instead of contracting effectively, impairing their ability to pump blood efficiently into the ventricles. Individuals with PAF often exhibit prolonged *P*-wave duration in electrocardiograms (ECGs), reflecting the disturbance in intra-atrial and interatrial conduction time^13^.

Hemochromatosis related to hereditary cause is called hereditary hemochromatosis (HH). HH is divided into various categories based on the presence or absence of the HFE gene mutation. The HFE-related category includes individuals who have either p.Cys282Tyr homozygosity or compound heterozygosity of p.Cys282Tyr with other rare HFE pathogenic variants or HFE deletion. However, this classification has low penetrance, and the presence of host-related or environmental factors should be taken into account when assessing iron overload. For individuals with other HFE genotypes, such as p.Cys282Tyr/His63Asp compound heterozygosity or p.His63Asp homozygosity, further genetic testing for rarer variants may be recommended. The non-HFE-related category encompasses rare pathogenic variants in genes other than HFE, including HJV-related, HAMP-related, TFR2-related, and SLC40A1 (GOF)-related genes^14^. While mutations in genes that regulate hepcidin could potentially be responsible, it is important to verify this through both functional experiments and population-based studies. The digenic classification refers to individuals who have mutations in two different genes involved in iron metabolism, which may include both HFE and non-HFE genes, either in a double heterozygous or double homozygous/heterozygous state. Finally, those undefined on a molecular level include patients for whom the molecular characterization is not available even after sequencing known genes, resulting in a provisional diagnosis^14^.

In HH, iron accumulation primarily occurs in parenchymal cells, while in transfusional hemochromatosis, it primarily accumulates in reticuloendothelial cells. The excess iron is stored as hemosiderin within these cells. Over time, this accumulation leads to cell death and fibrous replacement, resulting in the impairment or destruction of organ function. In the case of AF and hemochromatosis, the excessive iron accumulation in hemochromatosis can have implications for the development and progression of AF. While the primary sites of iron accumulation differ between hemochromatosis types (parenchymal cells in non-transfusional and reticuloendothelial cells in transfusional hemochromatosis), the end result is the same – the storage of excess iron as hemosiderin within cells.

This iron overload and subsequent hemosiderin deposition can lead to cell death and fibrous replacement over time. In the context of AF, these structural changes within the heart can contribute to the initiation and maintenance of the arrhythmia. The fibrous replacement and impairment of organ function caused by hemochromatosis can disrupt the normal electrical conduction system of the heart, creating a substrate that is conducive to the occurrence of AF. Furthermore, the increased iron levels in hemochromatosis may promote oxidative stress and inflammation, which are known to play a role in the pathophysiology of AF. These processes can further contribute to the electrical and structural remodeling of the atria, making them more susceptible to developing and sustaining AF episodes.

### Clinical presentation

Clinical presentation for AF can range from asymptomatic incidental findings on an ECG to a catastrophic stroke with an undiagnosed underlying irregular rhythm, which may be due to thicker viscosity of blood. More commonly, symptoms are similar to other arrhythmias like palpitations, dyspnea at rest or exertion, angina-like symptoms, fatigue or decreased exercise tolerance, lightheadedness, diaphoresis, dizziness, and pre-sync and syncope. AF can also masquerade as symptoms of heart failure exacerbation like weight gain, pedal edema, and pulmonary congestion^15^. Hemochromatosis can have a variable clinical presentation, and not all individuals with the condition will experience similar symptoms. However, common manifestations include fatigue, joint pain and stiffness, abdominal pain, skin pigmentation changes, loss of libido and impotence (in men), an increased risk of diabetes mellitus, cardiac symptoms such as shortness of breath and irregular heart rhythms, and the possibility of an enlarged liver or spleen. It is important to remember that symptoms can vary, and some individuals may not have any symptoms until the later stages of the disease^16^.

### Mechanisms of iron overload in hemochromatosis and its association with PAF

Iron overload can present in various forms, each with distinct underlying mechanisms. The main types of iron overload can be classified as follows:

1. Iron overload by excessive iron entry via enteral system; this type occurs due to hepcidin deficiency, a hormone responsible for regulating iron absorption. It includes two subtypes: hemochromatosis, which is caused by genetic defects leading to inadequate hepcidin production or function, and iron overload by acquired hepcidin deficiency, associated with abnormal red blood cell production^17^.
2. Iron overload by excessive parenteral entry of iron; this type is acquired and referred to as ‘iatrogenic’ iron overload, resulting from medical intervention. It consists of two subtypes: transfusional iron overload, which arises from repeated blood transfusions, and iron overload due to excessive parenteral iron supplementation through injections or infusions^18^.
3. Iron overload by genetic defect of cellular iron egress; this type is linked to ferroprotein disease, a condition affecting the transport of iron out of cells. Genetic mutations impair iron export, leading to intracellular accumulation^19^.
4. Iron overload with anemia due to genetic abnormalities of iron metabolism; this type is characterized by both anemia and excessive iron accumulation. It can be further divided into subcategories. The first subcategory involves genetic deficiencies in systemic iron metabolism, caused by mutations in specific genes involved in iron uptake, plasma iron transport, iron release from endosomes, cellular export of iron, and iron export from mitochondria. The second subcategory corresponds to defects in iron mitochondrial metabolism observed in congenital sideroblastic anemias^18^.

Most individuals with hemochromatosis exhibit elevated transferrin saturation (TfS), indicating iron overload. The accumulation of iron stores beyond normal levels is the main consequence of iron overload in hemochromatosis. The prevalence of organ damage resulting from iron overload remains a topic of debate, influenced by the bias in previous studies. Studies focused on individuals with severe complications or healthy blood donors may not provide a comprehensive picture. Population-based screening projects have shed more light on the issue. Studies in Norway showed increased liver iron stores in a significant number of homozygotes, but only a small percentage had moderate fibrosis or cirrhosis. In contrast, an Australian study reported a higher incidence of hemochromatosis-associated morbidity. These findings suggest that while a subset of homozygotes may experience complications, the overall risk remains uncertain^20–22^.

Iron overload, as seen in conditions such as hemochromatosis, has been suggested to have a potential association with PAF. While the exact mechanism linking iron overload to AF is not fully understood, several hypotheses have been proposed. One possible explanation is that the excess iron deposition in the heart tissue disrupts the normal electrical signaling and conduction pathways, leading to the development of AF. The presence of iron within the myocardium can alter the electrical properties of cardiac cells, affecting their excitability and refractoriness. This disturbance in the electrical system of the heart may create a substrate conducive to the initiation and maintenance of AF^20^.

Iron overload has also been linked to oxidative stress and inflammation, both of which are known to contribute to the development and progression of AF. Excess iron can promote the generation of reactive oxygen species (ROS) and increase oxidative stress in the myocardium. These ROS can cause cellular damage and impair the function of ion channels involved in cardiac electrical activity, potentially leading to arrhythmias like AF. Additionally, iron overload may trigger an inflammatory response within the heart, further promoting the remodeling of atrial tissue and increasing the risk of AF. Furthermore, iron overload can affect the structural integrity of the heart, including the myocardium and cardiac fibrosis. This structural remodeling can create an environment that facilitates the initiation and perpetuation of AF^21^.

It is important to note that while there is evidence suggesting a potential association between iron overload and PAF, more research is needed to fully understand the underlying mechanisms and establish a definitive link. If an individual has iron overload conditions such as hemochromatosis and experiences symptoms of AF, it is recommended to consult with a healthcare professional for proper evaluation, monitoring, and management of both conditions^22^.

## Prevalence and epidemiology

HH is the most common autosomal recessive disorder in whites, with a prevalence of 1 in 300–500 individuals^23^. HH types 2, 3, and 4 are seen worldwide, but type 1 is mostly seen in people of northern European descent^22^. The prevalence of hemochromatosis is the same in Europe, Australia, and other Western countries with excess in people of Celtic origin. It is less prevalent in patients of African descent. The white population has a six times higher risk of developing the disease than blacks. In hemochromatosis, men are affected around 2–3 times as often as women. The estimated ratio between men and women is 1.8:1 to 3:1. Women with hemochromatosis become symptomatic later in life than men due to the blood loss and consequent iron excretion associated with menstruation. In men, the disease usually becomes apparent in the fifth decade; however, in women, it presents in the sixth decade often. In contrast, juvenile hemochromatosis may appear in persons aged 10–30 years^24^.

Researchers of a 2010 study systematically reviewed 184 population-based studies for AF and estimated that ~33.5 million individuals in the world have AF^25^. AF prevalence has gradually increased over the last few decades. Based on the Centers for Disease Control and Prevention (CDC) estimate in 2017, 2.6–6.1 million people in the United States have AF. Further, the prevalence increases with age, and ~9% of all adults aged greater than 80 have AF^4,26^. Europe has a higher prevalence of AF compared to the United States. Similar to the prevalence, the incidence of AF increases with age. In all age groups, males are more commonly affected than females. Despite a high prevalence of risk factors, African Americans tend to have lower AF incidence compared to Caucasians^4^.

These epidemiological data suggest certain similarities and disparities in the prevalence and distribution of HH and AF. Both conditions are more prevalent in individuals of European descent and show a higher incidence with increasing age^4^. Additionally, gender differences exist, with men being more commonly affected. However, the prevalence of HH is influenced by ethnicity, while AF shows variations between regions. The lower incidence of AF in African Americans, despite shared risk factors, highlights potential racial disparities in the development of the condition^4^.

### Correlations between AF and hemochromatosis

The Genetics of Iron Status (GIS) consortium conducted a study to understand how specific genetic variations, known as single-nucleotide polymorphisms (SNPs), are associated with iron status. They analyzed data from multiple genome-wide association studies involving a large number of European individuals. In total, 48 972 participants (46.9% male) from 19 different cohorts were included in the study. Through their analysis, the researchers identified 12 SNPs that were significantly related to biomarkers of systemic iron status. These biomarkers include serum iron, TfS, ferritin, and transferrin levels^27^. Among these SNPs, five were associated with serum iron and TfS, six were associated with ferritin, and eight were associated with transferrin. These SNPs had a significant impact on systemic iron status, resulting in increased levels of serum iron, TfS, and ferritin, and decreased levels of transferrin^28^.

Three specific SNPs, namely rs1800562 and rs1799945 in the HFE gene, and rs855791 in the TMPRSS6 gene, consistently showed changes in all four biomarkers of systemic iron status. These SNPs played a major role in determining the differences observed in each iron status biomarker^29^. Due to their strong effects, these three SNPs were considered instrumental variables for iron status^30–32^. The statistical analyses performed indicated that the observed associations were unlikely to be biased by weak instrumental variables, as the calculated *F* statistic values ranged from 39 to 3340^33^. To obtain accurate results, the relationship between these SNPs and iron status biomarkers was adjusted for various factors, including age, principal component scores, and other study-specific covariates. The cohorts involved in the GIS consortium had a wide range of ages, from 14.8±2.2 to 68.2±15.5 years, with 53.1% of the participants being female. This study provides valuable insights into the genetic factors influencing iron status, demonstrating the significance of specific SNPs in determining systemic iron levels^34^. In this study, the researchers used a method called Mendelian randomization (MR) to investigate the association between systemic iron status biomarkers and the risk of AF. They aimed to overcome the limitations of observational studies by using genetic markers as instrumental variables to estimate causal effects. The MR analysis results showed that higher levels of serum iron, ferritin, and TfS were associated with an increased risk of AF. Conversely, higher levels of transferrin, which indicates decreased iron status, were associated with a lower probability of AF. The researchers used summary-level data from the largest meta-genome-wide association studies (GWAS) of European descent to minimize biases. They also performed various MR analysis methods and sensitivity analyses to ensure the robustness of their findings^34^. Iron overload has been associated with various metabolic disorders and cardiovascular diseases. The mechanisms by which iron status affects AF risk include oxidative stress, abnormal calcium ion handling, changes in cellular ion flow, and activation of nuclear factor-κB. Iron status can also influence atrial dilation and contribute to the development of AF^35^.

Although the research has some limitations, such as potential pleiotropic effects and a focus on European populations, the results provide evidence for a causal relationship between systemic iron status biomarkers and the risk of AF. By using MR analysis, this study contributes to our understanding of the role of iron status in the development of AF and suggests that iron status could be a promising clinical target for AF prevention and treatment^36^. Overall, these findings highlight the importance of considering iron status as a potential risk factor for AF and provide insights into the underlying mechanisms involved.

### Demographic factors

There are several demographic factors that affect the prevalence of AF.

1. Age and sex: Age and sex are significant factors in predicting the occurrence of AF. As individuals age, the prevalence of AF increases, with each decade doubling the risk. After accounting for age and other predisposing conditions, being male is associated with a 1.5-fold higher risk of developing AF^37–39^.
2. Hypertension: High blood pressure (hypertension) is a well-established risk factor for AF. Although the increase in risk is relatively modest (1.2–1.5 times), the high prevalence of hypertension in the general population makes it a significant contributing factor for AF, beyond age and sex. It is estimated that hypertension accounts for 14% of all AF cases^39,40^.
3. Valvular heart disease: Valvular heart disease is associated with an increased risk of AF, with men having a 1.8-fold higher risk and women having a 3.4-fold higher risk compared to those without valvular disease^39,40^.
4. Obesity: Overweight and obese individuals have an increased risk of AF compared to those with a normal body mass index (BMI). Overweight individuals have a 1.39–1.75 times higher risk, while obese individuals have a 1.99–2.35 times higher risk of developing AF^39,41^.
5. Alcohol: Excessive alcohol consumption is associated with an increased risk of AF. Binge drinking can trigger acute AF episodes, and heavy alcohol intake (≥36 g per day) is linked to a 1.34–1.46 times higher risk of new-onset AF^39,42,43^.
6. Smoking: Tobacco use has been recently linked to the development of AF, with a higher risk observed in individuals with a longer duration and higher intensity of smoking. Continued tobacco use is also associated with AF recurrence after catheter ablation^39,44^.
7. Physical activity/exercise: Regular moderate physical activity has multiple cardiovascular benefits and may reduce the incidence of AF. However, excessive or intense sports practices, such as endurance training, are associated with a higher prevalence of AF. Engaging in over 1500 hours of cumulative lifetime sports practice is linked to approximately a three-fold risk of developing AF and higher AF recurrence rates after catheter ablation^39,45^.

The demographic factors of hemochromatosis were evaluated by the Hemochromatosis and Iron Overload Screening (HEIRS) Study; it examined the influence of age, gender, and demographic factors on the occurrence of hemochromatosis. The study involved a large sample of 101 168 adults from diverse racial and ethnic backgrounds^46^. Here are the main findings:

1. Gender: The initial screening comprised 63 550 women (62.8%) and 37 618 men (37.2%). While the text does not explicitly state the prevalence of hemochromatosis by gender, it mentions that participants with specific TfS and serum ferritin (SF) values received further clinical evaluation, with different thresholds for men and women. This indicates a potential difference in prevalence between genders, although the exact details are not pr in the given information^46,47^.
2. Age: The median age of participants in the HEIRS Study was 50 years, ranging from 25 to 100 years. However, the specific impact of age on the prevalence of hemochromatosis is not elaborated upon in the text^46^.
3. Race/ethnicity: The study encompassed individuals from various racial and ethnic backgrounds. The prevalence of C282Y homozygosity, a genetic mutation associated with hemochromatosis, varied among different racial/ethnic groups. Non-Hispanic Caucasians exhibited a higher prevalence of C282Y homozygotes compared to Native Americans, Hispanics, African Americans, Pacific Islanders, and Asians. The study suggests that the effectiveness of HFE genotyping, used to identify C282Y homozygotes, may be lower in racial/ethnic groups other than non-Hispanic Caucasians^46,47^.
4. The HEIRS Study indicates that the prevalence of hemochromatosis, specifically C282Y homozygosity, is more common among non-Hispanic Caucasians compared to other racial/ethnic groups. However, the provided text does not offer detailed information regarding the specific impacts of age and gender on the prevalence of hemochromatosis^46^.

While the data do not directly establish a causal relationship between AF and hemochromatosis, the similarities in demographics, such as both conditions being more prevalent in individuals of European descent and males being more commonly affected, suggest a potential correlation. However, further research is needed to explore the specific relationship between these conditions and their shared demographic risk factors (Table 1).

**Table 1:** Demographic factors and their effects on AF and hemochromatosis.

Demographic factors	Influence on AF prevalence	Influence on hemochromatosis prevalence
Age	Increases with age	Not detailed in provided text
Sex	Male gender increases risk	Gender impact not detailed
Hypertension	Modest increase in risk	Not detailed in provided text
Valvular heart disease	Increased risk	Not detailed in provided text
Obesity	Increased risk for overweight	Not detailed in provided text
Alcohol	Excessive consumption linked to increased risk	Not detailed in provided text
Smoking	Linked to higher risk with longer and more intense smoking	Not detailed in provided text
Physical activity/exercise	Moderate exercise may reduce risk, excessive or intense sports practice may increase risk	Not detailed in provided text
Race/ethnicity	Not detailed in provided text	Prevalence varies among different racial/ethnic groups

## Pathophysiological link between PAF and hemochromatosis

The cause of AF is a complicated interaction between triggers and the underlying conditions. Triggers are responsible for initiating the arrhythmia, while the substrate sustains it^48,49^. The left atrial muscular sleeve extending into the pulmonary vein is the primary location for ectopic foci and trigger initiation in AF. AF can also be triggered by other arrhythmias such as Atrioventricular Reentrant Tachycardia (AVRT), atrial flutter, and premature atrial contractions (PACs). Atrial remodeling, which involves electrical, structural, and autonomic changes in the myocardium, promotes both the trigger and substrate mechanisms of AF. These changes result in altered sympathovagal activity, a shorter refractory period, and increased atrial inducibility, contributing to the propagation of AF^50,51^.

Inflammatory and oxidative changes are also noted at the molecular level, which can be correlated with the high C-reactive protein (CRP) level. AF can cause hemodynamic and electrophysiological changes causing increased susceptibility to new episodes of AF^52^. Oxidative stress can be brought on by iron overload, as seen in hemochromatosis; this is found to be the greatest correlating factor between the pathophysiology of both of these disorders. Oxidative stress can contribute to electrical and structural remodeling of the atria, which refers to changes in the heart’s electrical properties and physical structure. These remodeling processes make the atria more susceptible to developing and sustaining AF episodes. Electrical remodeling involves alterations in the electrical signaling pathways of the heart, leading to abnormal conduction and increased propensity for arrhythmias like AF. Structural remodeling refers to changes in the heart’s physical structure, such as fibrosis (excessive scar tissue formation) and dilation, which can disrupt the normal electrical conduction system and create a substrate for AF to occur and persist^52^.

The fibrous replacement refers to the formation of excessive scar tissue in place of the normal functional tissue. These structural changes can disrupt the normal architecture of the heart and impair its function. In the context of AF, these structural changes play a significant role. The fibrous replacement caused by hemochromatosis can disrupt the normal electrical conduction system of the heart. Scar tissue does not conduct electrical signals as effectively as healthy tissue, leading to altered pathways and conduction delays. These disruptions create an abnormal substrate within the heart, facilitating the occurrence and maintenance of AF. Additionally, the impaired organ function resulting from hemochromatosis can further contribute to the development of AF. The heart’s ability to pump blood efficiently may be compromised due to the fibrous replacement and overall damage caused by iron overload. This compromised function can lead to changes in blood flow patterns and contribute to the development of AF. Hemochromatosis can impact more than just organs, also affecting the endocrine system, joints, skin, and other tissues. Signs may encompass tiredness, joint discomfort, alterations in skin coloration, and hormonal irregularities^16^.

Hemochromatosis encompasses different types of the condition. Type 1 is the most common and inherited in an autosomal recessive manner, prevalent worldwide. Type 1 hemochromatosis is among the prevalent non-malignant blood conditions arising from an excess of iron. Both inherited and acquired factors can lead to hemochromatosis. The usual signs of type 1 hemochromatosis encompass diabetes, liver cirrhosis, and a bronze-like skin pigmentation, all stemming from an overabundance of iron in the body’s outer organs. However, these traditional symptoms are now infrequent, thanks to early genetic screening practices^53^. Types 2a and 2b are autosomal recessive disorders occurring in individuals of various ethnicities, with symptoms typically emerging in adolescence or early adulthood. Juvenile hemochromatosis, referred to as HH types 2a and 2b, arises from mutations in the HFE2 gene responsible for producing the hemojuvelin protein, and the HAMP gene responsible for encoding the iron-regulating hormone hepcidin^54^. Type 3 is an autosomal recessive disorder affecting individuals from different ethnic backgrounds, with symptoms manifesting in adulthood. Even though HH type 3 was initially identified with symptoms emerging in adulthood, there have been documented cases of individuals exhibiting juvenile onset. HH type 3 is an uncommon variation of HH distinguished by genetic changes in the Transferrin receptor 2 (TFR2) gene^16^. Type 4 is an autosomal dominant disease found in individuals of different ethnicities, and the age of onset can vary widely^16,53,54^. In HH type 4, individuals exhibit mutations in the SLC40A1 gene, which encodes the iron-exporting protein ferroportin, resulting in either the loss or gain of its functional capacity^16^.

The association between PAF and hemochromatosis can be understood by examining the underlying mechanisms of both conditions. Hemochromatosis, the most common cause being primary hemochromatosis, is an autosomal recessive condition where homozygotes with a mutation in the HFE protein experience increased iron absorption despite a normal dietary intake. The HFE protein regulates the production of hepcidin, an iron regulatory hormone produced by the liver^55^. Hepcidin determines the amount of iron absorbed from the diet and released from storage sites in the body. Abnormalities in the HFE gene, responsible for 90% of HH cases in individuals of Northern European descent, can lead to iron overload. Heterozygotes may show abnormalities in clinical markers of iron metabolism but do not acquire iron overload, although they have an increased risk of diabetes for unknown reasons^56^. Secondary hemochromatosis can be caused by conditions like erythropoietic hemochromatosis, where excess iron is absorbed due to the overproduction of red blood cells. This occurs in underlying red blood cell disorders or in individuals receiving chronic transfusions. Other conditions, such as porphyria cutanea tarda, can also lead to iron overload. Erythropoietic hemochromatosis is associated with the prevalence of the underlying disease and is found in a wider range of races compared to HH. Additionally, excessive iron consumption, whether from drinking beer prepared in steel drums or from accidental/intentional iron overdoses through dietary supplements, can cause hemochromatosis^57^.

Regarding the mechanism underlying AF, it involves a complex interaction between triggers and substrate. Triggers initiate the arrhythmia, while substrate maintains it^48^. Afterdepolarization induced by the action potential can cause extrasystole, but it cannot sustain persistent arrhythmia. However, impulses from these extrasystoles, discharged at high frequency, encounter variable excitability or refractoriness in the myocardium, leading to functional electrical blocks. This results in the formation of re-entry circuits that maintain persistent arrhythmia^49^. Ectopic foci and trigger origin are often found in the left atrial muscular sleeve extending into the pulmonary vein, where myocardial cells have a shorter refractory period compared to surrounding myocardium. Atrial remodeling, caused by electrical, structural, and autonomic changes in the myocardium, promotes both triggers and substrates for AF^49^. Inflammatory and oxidative changes at the molecular level, along with increased CRP levels, are also observed. Hemodynamic and electrophysiological changes caused by AF contribute to increased susceptibility to new episodes of AF^52^.

The association between PAF and hemochromatosis can be attributed to the underlying mechanisms of each condition. Hemochromatosis involves mutations in the HFE protein, leading to iron overload, while AF is characterized by triggers and substrates that initiate and maintain the arrhythmia. Understanding these mechanisms can provide insights into the relationship between the two conditions.

### Role of iron overload in promoting cardiac arrhythmias

The role of iron overload in promoting cardiac arrhythmias is still not fully understood, and there are several factors that complicate the understanding of its etiology. One complicating factor is the heterogeneous deposition of iron in cardiomyocytes in individuals with iron overload, making it unclear if this accumulation pattern universally alters iron channels in the entire cardiomyocyte. Furthermore, it is uncertain whether iron overload itself, heart failure, elevated oxidative stress, or a combination of these factors is the primary cause of arrhythmias and conduction abnormalities^58^. Elevated oxidative stress, which can be induced by both iron overload and heart failure, has been shown to affect the function of ion channels, including the L-type calcium channel (ICa-L) and late sodium currents (INa-L). Oxidative stress can also affect mitochondrial functions. These alterations in ion channels and mitochondrial functions may contribute to arrhythmias. However, the exact etiologic role of oxidative stress in arrhythmias related to iron overload has not been conclusively proven. Notably, the effects of oxidative stress on L-type calcium channels may counteract the reported decreased activity of these channels in iron overload, leading to conflicting results regarding arrhythmogenicity^59^. Elevated oxidative stress can also inhibit the KATP channel and downregulate Ito, Ikr, and Iks channels, potentially contributing to oxidative stress-induced arrhythmias. Furthermore, it can increase the activity of the Na+–Ca2+ exchanger (NCX), leading to afterdepolarization and the development of arrhythmias. Animal studies have demonstrated the etiologic role of elevated oxidative stress in ischemia reperfusion-associated arrhythmias^60^. In human studies, an association between elevated oxidative stress and AF has been observed, particularly in the postsurgical period^61^. This is interesting because AF is also associated with iron overload in humans, where oxidative stress is elevated^62^. In addition to oxidative stress, heart failure itself can contribute to arrhythmias through various mechanisms, including downregulation of potassium currents (Ito, Ikr, Iks, and Ik1), an increase in late sodium current, defective calcium sequestration, upregulation of calcium extrusion via NCX, and alterations in gap junctions. Enhanced delayed calcium leak through the ryanodine receptor2, leading to delayed afterdepolarization, has also been implicated as an arrhythmogenic mechanism in heart failure^63^. Translating findings from cellular experiments to whole animal experiments is an area that requires further advancement in the study of iron overload-induced arrhythmias. More advanced pharmacological and molecular approaches are needed to better understand the arrhythmogenicity of iron overload and its management.

### Impact of hemochromatosis on cardiac structure and function

Hemochromatosis can show up as heart problems due to having too much iron. This can lead to issues like abnormal heart rhythms, high blood pressure in the lungs, and a type of heart failure called congestive heart failure. The heart problems in hemochromatosis follow a pattern – they start with stiffness in the heart’s muscle and, if not treated, can turn into a type where the heart does not pump effectively. As the heart problems worsen, it can lead to congestive heart failure. Too much iron in the heart causes harmful substances to form and this hurts the heart cells, making the heart’s job harder^64^.

## Comparison between hemochromatosis (HFE) and PAF/AF

Table 2.

**Table 2:** Comparison between HFE-hemochromatosis and PAF/AF.

Aspect	Hemochromatosis (HFE)	Paroxysmal atrial fibrillation (PAF/AF)
Definition	Autosomal recessive condition	Form of atrial fibrillation
Cause/mechanism	HFE gene mutations, iron overload	Triggers and substrate interaction
Types	Types 1, 2a, 2b, 3, 4	N/A
Onset age	Varies (depending on type)	Can occur at any age
Primary location	Iron overload in various organs	Left atrial muscular sleeve extending into pulmonary vein
Remodeling	Electrical, structural, and autonomic changes	Electrical, structural, and autonomic changes
Inflammation/oxidative stress	High C-reactive protein (CRP) levels	Correlated with high CRP levels
Associated conditions	Diabetes, liver cirrhosis, bronze-like skin pigmentation	N/A
Treatment	Phlebotomy, chelation therapy (in specific cases), liver transplantation	Antithrombotic therapy, rate control, rhythm control, ablation
Prognosis	Improves with early diagnosis and treatment	Associated with increased cardiovascular mortality

## Treatment strategies

The current treatment options for PAF include antithrombotic therapy, rate control, and rhythm control. Antithrombotic therapy is used to reduce the risk of stroke in PAF patients. The selection of antithrombotic therapy is based on the patient’s individualized stroke risk, assessed using the CHA2DS2-VASc risk model^65^. Warfarin and non-vitamin K antagonist oral anticoagulants (NOACs) have shown similar effectiveness in reducing stroke risk. The choice of antithrombotic agent depends on factors such as risk factors, cost, tolerability, patient preference, and potential drug interactions^66^. Rate control is the preferred strategy for asymptomatic PAF patients. Medications like beta-blockers, calcium channel blockers, or digoxin are used to control the heart rate. The goal of rate control is to achieve an optimal heart rate; however, guidelines on target heart rate are lacking. In patients with chronic heart failure, carvedilol therapy has shown significant improvement in left ventricular function^67^. Rhythm control, achieved through pharmacological therapy or non-pharmacological methods like direct current cardioversion (DCCV) or catheter ablation, is considered based on symptoms, age, and comorbidities. Class I antiarrhythmics should be avoided in patients with a history of coronary artery disease or heart failure. Class III antiarrhythmics, like amiodarone, have greater long-term efficacy but may cause toxicities. DCCV is commonly performed in patients with hemodynamic compromise or new-onset PAF. Anticoagulation is recommended before and after DCCV. Catheter ablation has high success rates and can be considered for patients refractory to medical therapy^68^.

The current treatment options for hemochromatosis primarily involve phlebotomy, which is the process of drawing off red blood cells to reduce iron levels in the body. Phlebotomy is usually performed once or twice a week, and patients may require 50–100 phlebotomies to normalize iron levels. Once iron levels have stabilized, lifelong but less frequent phlebotomy (typically 3–4 times a year) is necessary to maintain iron balance and achieve a ferritin level of less than 50 mcg/l^69^. Phlebotomy has been shown to improve insulin sensitivity, skin pigmentation, and fatigue in hemochromatosis patients, although it may not reverse preexisting end-organ damage such as cirrhosis, hypogonadism, or arthropathy^69^. Alcohol consumption should be strictly avoided in hemochromatosis patients as it can accelerate liver and pancreatic toxicity. Treatment for associated end-organ dysfunction, such as insulin therapy for pancreatic dysfunction, is indicated. Early detection and treatment of hemochromatosis can prevent the development of end-organ dysfunction and improve long-term outcomes. However, if severe end-organ damage has already occurred, the prognosis may be poor, and patients may have a reduced life expectancy^70^. Chelation therapy, which involves the use of iron-chelating agents to remove excess iron, is more beneficial in erythropoietic hemochromatosis than in HH where phlebotomy is typically the preferred treatment. Intravenous iron-chelating agent deferoxamine and oral iron chelators like deferasirox and deferiprone are effective in mobilizing and excreting iron^71^. In some cases, erythropoietin may be administered along with phlebotomy to maintain hemoglobin concentration while promoting iron mobilization. Liver transplantation may be considered for patients with end-stage liver disease associated with hemochromatosis. However, studies have shown that patients with iron overload disorders, including hemochromatosis, who undergo liver transplantation have lower survival rates compared to those without hemochromatosis-related causes^72^.

Managing PAF in individuals with hemochromatosis presents challenges due to the need for balancing treatment strategies for both conditions. Phlebotomy is the primary treatment for hemochromatosis and can worsen anemia, which is commonly seen in these patients. Strict alcohol prohibition is necessary to prevent liver and pancreatic toxicity. The impact of PAF on hemochromatosis and vice versa requires careful consideration, as end-organ damage may limit treatment options and prognosis. Individualized management plans should address the specific challenges and risks associated with managing both conditions simultaneously. Currently, there are no specific emerging therapies or interventions that simultaneously target both PAF and hemochromatosis. The focus remains on treating each condition individually and optimizing their respective treatment strategies. Further research is needed to explore potential synergistic effects or tailored approaches to managing these conditions together. Currently, there are no specific emerging therapies or interventions that simultaneously target both hemochromatosis and PAF. The focus remains on managing each condition individually and optimizing their respective treatment strategies. Further research is needed to explore potential synergistic effects or tailored approaches to managing both conditions simultaneously.

## Prognosis and outcomes

If left untreated, hemochromatosis can lead to progressive damage of the liver and lead to cirrhosis, hepatocellular carcinoma, and other complications associated with iron overload in the tissues and organs^73^. The prognosis of hemochromatosis, especially if left untreated, can have implications for the risk of developing AF. Hemochromatosis is characterized by excessive iron accumulation in various organs, including the heart. If not managed appropriately, this iron overload can lead to significant damage and dysfunction of the affected organs. With advances in diagnosis and management of this condition, the prognosis has improved in the last few decades. Hepatic fibrosis or cirrhosis is the main prognostic indicator at the time of diagnosis. Early diagnosis and regular treatment with phlebotomy can decrease most of the complications associated with hemochromatosis^74^. PAF is associated with increased cardiovascular mortality^75^. Anticoagulation in all patients with a CHA2DS2-VASc score equal to 2 has shown an improved prognosis. Patients not on anticoagulation are at high risk for thromboembolic events. Fifty-five percent of AF patients are not on antithrombotic therapy, which accounts for over 50 000 strokes annually^76^. The prognosis for AF-related stroke is worse compared to non-AF-related cerebrovascular events. Patients with heart failure are at increased risk of developing AF-related complications. Elevated high-sensitivity CRP (hs-CRP) and troponins have been implicated with adverse outcomes in AF patients ^80^. In the context of AF, the structural changes and impairments caused by hemochromatosis can disrupt the normal electrical conduction system of the heart. Fibrous replacement and organ dysfunction resulting from hemochromatosis create a substrate that is conducive to the occurrence and maintenance of AF. These changes make the heart more vulnerable to developing arrhythmias, including AF.

## Results

### Mechanisms of iron overload in hemochromatosis

Hemochromatosis is characterized by iron accumulation primarily in parenchymal cells, leading to cell death and fibrous replacement. It can be caused by genetic defects, acquired hepcidin deficiency, transfusional iron overload, or genetic defects in cellular iron egress.

### Prevalence and epidemiology

HH is the most common autosomal recessive disorder in whites. Different types of hemochromatosis are seen worldwide, with type 1 being more prevalent in people of northern European descent. The prevalence of hemochromatosis varies among racial/ethnic groups.

### Correlations between AF and hemochromatosis

Genetic variations associated with iron status biomarkers have been found to be associated with an increased risk of AF. Higher levels of serum iron, ferritin, and transferrin saturation are linked to an increased risk of AF, while higher levels of transferrin, indicating decreased iron status, are associated with a lower probability of AF.

### Demographic factors

Age, sex, hypertension, valvular heart disease, obesity, excessive alcohol consumption, smoking, and physical activity/exercise are factors that influence the prevalence of AF. The impact of age, gender, and demographic factors on the occurrence of hemochromatosis has been studied, with non-Hispanic Caucasians showing a higher prevalence of certain genetic mutations associated with hemochromatosis.

### Pathophysiological link

The association between PAF and hemochromatosis can be attributed to the underlying mechanisms of each condition. Hemochromatosis involves mutations in the HFE protein, leading to iron overload, while AF is characterized by triggers and substrates that initiate and maintain the arrhythmia. Understanding these mechanisms provides insights into the relationship between the two conditions.

### Role of iron overload

Iron overload in hemochromatosis may promote cardiac arrhythmias, although the exact mechanisms are not fully understood. Elevated oxidative stress, influenced by iron overload and heart failure, can affect ion channels and mitochondrial functions, potentially contributing to arrhythmias. However, the specific etiologic role of oxidative stress in arrhythmias related to iron overload requires further investigation.

### Impact on cardiac structure and function

Hemochromatosis can lead to iron overload cardiomyopathy, arrhythmias, pulmonary hypertension, and congestive heart failure. Iron deposition in the heart generates ROS, damaging cardiomyocytes and impairing cardiac function.

### Clinical manifestations and diagnosis

Clinical signs of hemochromatosis depend on the affected organ system, while PAF commonly presents with palpitations, chest pain, and reduced exercise tolerance. The diagnostic process for hemochromatosis involves assessing iron markers through blood tests, genotypic testing for specific HFE gene mutations, and considering the need for a liver biopsy in certain cases. The diagnosis of AF includes a thorough history, physical examination, and characteristic ECG findings.

### Treatment strategies

Treatment for hemochromatosis primarily involves phlebotomy to reduce iron levels. Antithrombotic therapy, rate control, and rhythm control are treatment options for PAF. The selection of treatment depends on individualized factors such as stroke risk, symptoms, age, and comorbidities.

### Prognosis and outcomes

Untreated hemochromatosis can lead to progressive liver damage, cirrhosis, and associated complications. Early diagnosis and regular treatment with phlebotomy improve the prognosis. PAF is associated with increased cardiovascular mortality, and anticoagulation is recommended to reduce the risk of thromboembolic events. The prognosis for AF-related stroke is worse compared to non-AF-related cerebrovascular events, and heart failure patients are at increased risk of AF-related complications (Table 3).

**Table 3:** Results concluded by this paper.

Mechanisms of iron overload in hemochromatosis	• Hemochromatosis causes iron buildup in cells, leading to cell death and fibrous replacement. • It can result from genetic defects, hepcidin deficiency, transfusional iron overload, or cellular iron egress issues.
Prevalence and epidemiology	• Hereditary hemochromatosis is common in white populations. • Type 1 is more common in people of northern European descent. • Prevalence varies among different racial and ethnic groups.
Correlations between AF and hemochromatosis	• Genetic variations linked to iron levels are associated with increased AF risk. • High serum iron, ferritin, and transferrin saturation raise AF risk. • Higher transferrin levels (indicating lower iron status) lower the likelihood of AF.
Demographic factors	• Age, sex, hypertension, valvular heart disease, obesity, alcohol, smoking, and physical activity influence AF prevalence. • Non-Hispanic Caucasians have a higher prevalence of certain genetic mutations related to hemochromatosis.
Pathophysiological link	• Hemochromatosis involves mutations in the HFE protein, leading to iron overload. • AF is characterized by triggers and substrates that initiate and sustain the arrhythmia.
Role of iron overload	• Iron overload in hemochromatosis may contribute to cardiac arrhythmias, possibly through elevated oxidative stress. • Oxidative stress can impact ion channels and mitochondrial functions, potentially leading to arrhythmias.
Impact on cardiac structure and function	• Hemochromatosis can lead to iron overload cardiomyopathy, arrhythmias, pulmonary hypertension, and congestive heart failure. • Iron buildup in the heart generates reactive oxygen species, damaging heart cells and impairing function.
Clinical manifestations and diagnosis	• Hemochromatosis symptoms depend on the affected organ system. • Paroxysmal AF commonly presents with palpitations, chest pain, and reduced exercise tolerance. • Hemochromatosis diagnosis involves blood tests for iron markers, genotypic testing for specific HFE gene mutations, and sometimes a liver biopsy. • AF diagnosis includes a thorough history, physical examination, and characteristic electrocardiogram (ECG) findings.
Treatment strategies	• Hemochromatosis treatment primarily involves phlebotomy to reduce iron levels. • Paroxysmal AF can be managed with antithrombotic therapy, rate control, and rhythm control, based on individualized factors.
Prognosis and outcomes	• Untreated hemochromatosis can lead to progressive liver damage, cirrhosis, and associated complications. • Early diagnosis and regular phlebotomy treatment improve the prognosis. • Paroxysmal AF is associated with increased cardiovascular mortality, and anticoagulation is recommended to reduce thromboembolic risks. • AF-related strokes have a worse prognosis compared to non-AF-related cerebrovascular events. Heart failure patients are at increased risk of AF-related complications.

## Conclusion

PAF is characterized by intermittent irregular heartbeats. Hemochromatosis, an excess iron condition, is linked to AF due to its impact on heart structure and function. Age, gender, and certain conditions influence both AF and hemochromatosis. Understanding their shared mechanisms is crucial. Iron overload in hemochromatosis may contribute to heart rhythm issues via oxidative stress, although details are unclear. Hemochromatosis causes heart problems, from muscle dysfunction to rhythm disturbances and heart failure. Diagnosis involves blood tests, genetic testing, and, in some cases, a liver biopsy. Treatment includes reducing iron levels through phlebotomy. Early intervention improves hemochromatosis outcomes. Untreated hemochromatosis leads to liver damage, cirrhosis, and complications. PAF raises cardiovascular mortality risk, warranting anticoagulation. AF-related strokes have a worse prognosis, particularly in heart failure patients. Research is ongoing for better management strategies. In our opinion, it is imperative that these two conditions be looked at collectively on a more detailed level so that more inclusive treatment strategies can be devised. We believe that the complex interplay between PAF and hemochromatosis underscores the need for an integrative approach to diagnosis and treatment. Timely intervention, guided by a thorough understanding of shared mechanisms, will undoubtedly yield significant clinical benefits for affected individuals.

## Ethical approval

Ethics approval was not required for this review.

## Consent

Informed consent was not required for this review.

## Sources of funding

No funding received.

## Author contribution

G.A. and S.R.: designed the study and conceived the idea; H.K.S., M.M., and M.H.W., K.K.: wrote the first draft; H.F., N.M.A, K.M., and V.K.: wrote the second draft; U.T. and N.R.: worked on the revisions and supervised the whole manuscript. All authors equally contributed to this work.

## Conflicts of interest disclosure

No conflicts of interest were declared.

## Research registration unique identifying number (UIN)

It is not a human study.

## Guarantor

Koushik Majumder.

## Data availability statement

Data sharing is not applicable to this article.

## Provenance and peer review

Not commissioned, externally peer-reviewed.

## References

[1] Jabre P Roger VL Murad MH. Mortality associated with atrial fibrillation in patients with myocardial infarction: a systematic review and meta-analysis. Circulation 2011;123:1587–1593. 21464054 10.1161/CIRCULATIONAHA.110.986661 PMC3082773

[2] Muthusamy S Ramesh SS Singh Z. Paroxysmal atrial fibrillation in young and middle-aged hypertensive patients: a cross-sectional study. J Assoc Physicians India 2016;64:18–22.

[3] Proietti M Lane DA Boriani G. Challenges and advances in the management of atrial fibrillation in patients with chronic kidney disease. Clin Kidney J 2020;13:750–760.

[4] Go AS Hylek EM Phillips KA. Prevalence of diagnosed atrial fibrillation in adults: national implications for rhythm management and stroke prevention: the AnTicoagulation and Risk Factors in Atrial Fibrillation (ATRIA) Study. JAMA 2001;285:2370–5. 11343485 10.1001/jama.285.18.2370

[5] Zoni-Berisso M Lercari F Carazza T. Epidemiology of atrial fibrillation: European perspective. Clin Epidemiol 2014;6:213–220. 24966695 10.2147/CLEP.S47385 PMC4064952

[6] Hart RG Pearce LA Aguilar MI. Meta-analysis: antithrombotic therapy to prevent stroke in patients who have nonvalvular atrial fibrillation. Ann Intern Med 2007;146:857–67. 17577005 10.7326/0003-4819-146-12-200706190-00007

[7] Zhang C Kasner SE. Paroxysmal atrial fibrillation in cryptogenic stroke: an overlooked explanation? Curr Atheroscler Rep 2015;17:66. 26486510 10.1007/s11883-015-0547-0

[8] Lucotte G. Celtic origin of the C282Y mutation of hemochromatosis. Blood Cells Mol Dis 1998;24:433–8. 9851897 10.1006/bcmd.1998.0212

[9] Adams PC. Epidemiology and diagnostic testing for hemochromatosis and iron overload. Int J Lab Hematol 2015;37:25–30. 25976957 10.1111/ijlh.12347

[10] Bacon BR Adams PC Kowdley KV. Diagnosis and management of hemochromatosis: 2011 practice guideline by the American Association for the Study of Liver Diseases. Hepatology 2011;54:328–43. 21452290 10.1002/hep.24330 PMC3149125

[11] Edwards CQ Cartwright GE Skolnick MH. Homozygosity for hemochromatosis: clinical manifestations. Ann Intern Med 1980;93:519–25. 7436183 10.7326/0003-4819-93-4-519

[12] Niederau C Fischer R Sonnenberg A. Survival and causes of death in cirrhotic and in noncirrhotic patients with primary hemochromatosis. N Engl J Med 1985;313:1256–62. 4058506 10.1056/NEJM198511143132004

[13] Baranchuk A Simpson CS Methot M. Paroxysmal atrial fibrillation: 30 years later. Can J Cardiol 2001;17:1287–94.

[14] Grosse R Kaeser T Legati A. Hemochromatosis classification update and diagnostic algorithms: expert opinions from the Second World Congress on Iron Metabolism. Blood 2017;139:3018–3036.

[15] HFE-related hereditary hemochromatosis. In: Adam MP, Ardinger HH, Pagon RA, et al., editors. GeneReviews^®^. University of Washington, Seattle; 1993–2022.

[16] National Center for Biotechnology Information (US). Hemochromatosis. In: Adam MP, Ardinger HH, Pagon RA, *et al*., editors. GeneReviews^®^. University of Washington, Seattle; 2003.

[17] Brissot P Pietrangelo A Adams PC. Haemochromatosis. Nat Rev Dis Primers 2018;4:18016. 29620054 10.1038/nrdp.2018.16 PMC7775623

[18] Brissot P Troadec M-B Loréal O. Pathophysiology and classification of iron overload diseases; update 2018. Transfus Clin Biol 2018;26:80–88. 30173950 10.1016/j.tracli.2018.08.006

[19] LeLan C Mosser A Ropert M. Sex and acquired cofactors determine phenotypes of ferroportin disease. Gastroenterology 2011;140:1199–207.e1–2. 21199650 10.1053/j.gastro.2010.12.049

[20] Ajioka RS Kushner JP. Clinical consequences of iron overload in hemoglobinopathies and other conditions. Blood 2003;101:3351–3353. 12707221 10.1182/blood-2002-11-3453

[21] Asberg AH Hveem K Thorstensen K. Screening for hemochromatosis: high prevalence and low morbidity in an unselected population of 65,238 persons. Scand J Gastroenterol 2001;36:1108–15. 11589387 10.1080/003655201750422747

[22] Olynyk JK Cullen DJ Aquilia S. A population-based study of the clinical expression of the hemochromatosis gene. N Engl J Med 1999;341:718–24. 10471457 10.1056/NEJM199909023411002

[23] Adams PC. Epidemiology and diagnostic testing for hemochromatosis and iron overload. Int J Lab Hematol 2015;37(Suppl 1):25–30. 25976957 10.1111/ijlh.12347

[24] National Center for Biotechnology Information. Iron overload and hemochromatosis. In: Adam MP, Ardinger HH, Pagon RA, *et al*., editors. GeneReviews^®^. University of Washington, Seattle; 2000.

[25] Chugh SS Havmoeller R Narayanan K. Worldwide epidemiology of atrial fibrillation: a Global Burden of Disease 2010 Study. Circulation 2014;129:837–47. 24345399 10.1161/CIRCULATIONAHA.113.005119 PMC4151302

[26] Ball J Carrington MJ McMurray JJ. Atrial fibrillation: profile and burden of an evolving epidemic in the 21st century. Int J Cardiol 2013;167:1807–24. 23380698 10.1016/j.ijcard.2012.12.093

[27] Benyamin B Esko T Ried JS. Novel loci affecting iron homeostasis and their effects in individuals at risk for hemochromatosis. Nat Commun 2014;5:4926. 25352340 10.1038/ncomms5926 PMC4215164

[28] Storey JA Connor RF Lewis ZT. The transplant iron score as a predictor of stem cell transplant survival. J Hematol Oncol 2009;2:44. 19852846 10.1186/1756-8722-2-44 PMC2770452

[29] Breitling LP Koenig W Fischer M. Type II secretory phospholipase A2 and prognosis in patients with stable coronary heart disease: Mendelian randomization study. PLoS One 2011;6:e22318. 21799821 10.1371/journal.pone.0022318 PMC3142130

[30] Gill D Del Greco MF Walker AP. The effect of iron status on risk of coronary artery disease. Arterioscler Thromb Vasc Biol 2017;37:1788–92. 28684612 10.1161/ATVBAHA.117.309757

[31] Gill D Brewer CF Monori G. Effects of genetically determined iron status on risk of venous thromboembolism and carotid atherosclerotic disease: a Mendelian randomization study. J Am Heart Assoc 2019;8:e012994. 31310728 10.1161/JAHA.119.012994 PMC6761644

[32] Gill D Monori G Tzoulaki I. Iron status and risk of stroke. Stroke 2018;49:2815–21. 30571402 10.1161/STROKEAHA.118.022701 PMC6257507

[33] Huang L Li L Luo X. The association between serum iron status and risk of asthma: a 2-sample Mendelian randomization study in descendants of Europeans. Am J Clin Nutr 2019;110:959–68. 31380560 10.1093/ajcn/nqz162

[34] Wang T Cheng J Wang Y. Genetic support of a causal relationship between iron status and atrial fibrillation: a Mendelian randomization study. Genes Nutr 2022;17:8. 35637428 10.1186/s12263-022-00708-9 PMC9153204

[35] Gao G Dudley SC Jr. Redox regulation, NF-κB, and atrial fibrillation. Antioxid Redox Signal 2009;11:2265–2277. 19309257 10.1089/ars.2009.2595 PMC2819799

[36] Holmes MV Ala-Korpela M Smith GD. Mendelian randomization in cardiometabolic disease: challenges in evaluating causality. Nat Rev Cardiol 2017;14:577–90. 28569269 10.1038/nrcardio.2017.78 PMC5600813

[37] Ohsawa M Okayama A Sakata K. Rapid increase in estimated number of persons with atrial fibrillation in Japan: an analysis from national surveys on cardiovascular diseases in 1980, 1990 and 2000. J Epidemiol 2005;15:194–196. 16195640 10.2188/jea.15.194 PMC7904304

[38] Piccini JP Hammill BG Sinner MF. Incidence and prevalence of atrial fibrillation and associated mortality among Medicare beneficiaries, 1993–2007. Circ Cardiovasc Qual Outcomes 2012;5:85–930. 22235070 10.1161/CIRCOUTCOMES.111.962688 PMC3332107

[39] Andrade J Khairy P Dobrev D. The clinical profile and pathophysiology of atrial fibrillation: relationships among clinical features, epidemiology, and mechanisms. Circ Res 2014;114:1453–1468. 24763464 10.1161/CIRCRESAHA.114.303211

[40] Kannel WB Wolf PA Benjamin EJ. Prevalence, incidence, prognosis, and predisposing conditions for atrial fibrillation: population-based estimates. Am J Cardiol 1998;82:2N–9N. 10.1016/s0002-9149(98)00583-9 9809895

[41] Dublin S French B Glazer NL. Risk of new-onset atrial fibrillation in relation to body mass index. Arch Intern Med 2006;166:2322–2328. 17130384 10.1001/archinte.166.21.2322

[42] Frost L Vestergaard P. Alcohol and risk of atrial fibrillation or flutter: a cohort study. Arch Intern Med 2004;164:1993–1998. 15477433 10.1001/archinte.164.18.1993

[43] Mukamal KJ Tolstrup JS Friberg J. Alcohol consumption and risk of atrial fibrillation in men and women: the Copenhagen City Heart Study. Circulation 2005;112:1736–1742. 16157768 10.1161/CIRCULATIONAHA.105.547844

[44] Chamberlain AM Agarwal SK Folsom AR. Smoking and incidence of atrial fibrillation: results from the Atherosclerosis Risk in Communities (ARIC) study. Heart Rhythm 2011;8:1160–1166. 21419237 10.1016/j.hrthm.2011.03.038 PMC3139831

[45] Mozaffarian D Furberg CD Psaty BM. Physical activity and incidence of atrial fibrillation in older adults: the cardiovascular health study. Circulation 2008;118:800–807. 18678768 10.1161/CIRCULATIONAHA.108.785626 PMC3133958

[46] McLaren CE Barton JC Adams PC. Hemochromatosis and Iron Overload Screening (HEIRS) study design for an evaluation of 100,000 primary care-based adults. Am J Med Sci 2003;325:53–62. 12589228 10.1097/00000441-200302000-00001

[47] McLaren GD Gordeuk VR. Hereditary hemochromatosis: insights from the Hemochromatosis and Iron Overload Screening (HEIRS) Study. Hematology Am Soc Hematol Educ Program 2009;2009:195–206. 10.1182/asheducation-2009.1.195 PMC3829617 20008199

[48] Nattel S Dobrev D. The multidimensional role of calcium in atrial fibrillation pathophysiology: mechanistic insights and therapeutic opportunities. Eur Heart J 2012;33:1870–7. 22507975 10.1093/eurheartj/ehs079

[49] Wakili R Voigt N Kääb S. Recent advances in the molecular pathophysiology of atrial fibrillation. J Clin Invest 2011;121:2955–68. 21804195 10.1172/JCI46315 PMC3148739

[50] Wijffels MC Kirchhof CJ Dorland R. Atrial fibrillation begets atrial fibrillation. A study in awake chronically instrumented goats. Circulation 1995;92:1954–68. 7671380 10.1161/01.cir.92.7.1954

[51] Li D Fareh S Leung TK. Promotion of atrial fibrillation by heart failure in dogs: atrial remodeling of a different sort. Circulation 1999;100:87–95. 10393686 10.1161/01.cir.100.1.87

[52] Nattel S. Atrial electrophysiological remodeling caused by rapid atrial activation: underlying mechanisms and clinical relevance to atrial fibrillation. Cardiovasc Res 1999;42:298–308. 10533568 10.1016/s0008-6363(99)00022-x

[53] Yun S Vincelette ND. Update on iron metabolism and molecular perspective of common genetic and acquired disorder, hemochromatosis. Crit Rev Oncol Hematol 2015;95:12–25. 25737209 10.1016/j.critrevonc.2015.02.006

[54] Bardou-Jacquet E Brissot P. Diagnostic evaluation of hereditary hemochromatosis (HFE and non-HFE). Hematol Oncol Clin North Am 2014;28:625–35. 25064704 10.1016/j.hoc.2014.04.006

[55] Means RT. Hepcidin and iron regulation in health and disease. Am J Med Sci 2013;345:57–60. 22627267 10.1097/MAJ.0b013e318253caf1 PMC3430792

[56] Bulaj ZJ Griffen LM Jorde LB. Clinical and biochemical abnormalities in people heterozygous for hemochromatosis. N Engl J Med 1996;335:1799–805. 8943161 10.1056/NEJM199612123352403

[57] Pietrangelo A. Hereditary hemochromatosis: pathogenesis, diagnosis, and treatment. Gastroenterology 2010;139:393–408. 20542038 10.1053/j.gastro.2010.06.013

[58] Shizukuda Y Rosing DR. Iron overload and arrhythmias: influence of confounding factors. J Arrhythm 2019;35:575–583. 31410226 10.1002/joa3.12208 PMC6686354

[59] Sovari AA. Cellular and molecular mechanisms of arrhythmia by oxidative stress. Cardiol Res Pract 2016;2016:9656078. 26981310 10.1155/2016/9656078 PMC4770129

[60] Dhalla NS Elmoselhi AB Hata T. Status of myocardial antioxidants in ischemia‐reperfusion injury. Cardiovasc Res 2000;47:446–56. 10963718 10.1016/s0008-6363(00)00078-x

[61] Lubbers ER Murphy NP Mohler PJ. Defining the links between oxidative stress‐based biomarkers and postoperative atrial fibrillation. J Am Heart Assoc 2015;4:e002110. 25994438 10.1161/JAHA.115.002110 PMC4599434

[62] Shizukuda Y Bolan CD Tripodi DJ. Does oxidative stress modulate left ventricular diastolic function in asymptomatic subjects with hereditary hemochromatosis? Echocardiography 2009;26:1153–8. 19725855 10.1111/j.1540-8175.2009.00956.x PMC3397801

[63] Yano M Yamamoto T Ikeda Y. Mechanisms of disease: Ryanodine receptor defects in heart failure and fatal arrhythmia. Nat Clin Pract Cardiovasc Med 2006;3:43–52. 16391617 10.1038/ncpcardio0419

[64] Moir HJ Stewart G Brown LA. Exercise-induced oxidative stress and its implications for skeletal muscle health. Free Radic Res 2021;55:489–504. doi:10.1080/10715762.2021.1891833

[65] Friberg L Rosenqvist M Lip GY. Evaluation of risk stratification schemes for ischaemic stroke and bleeding in 182 678 patients with atrial fibrillation: the Swedish Atrial Fibrillation cohort study. Eur Heart J 2012;33:1500–10. 22246443 10.1093/eurheartj/ehr488

[66] Connolly SJ Ezekowitz MD Yusuf S RE-LY Steering Committee and Investigators. Dabigatran versus warfarin in patients with atrial fibrillation. N Engl J Med 2009;361:1139–51. 19717844 10.1056/NEJMoa0905561

[67] Granger CB Alexander JH McMurray JJ ARISTOTLE Committees and Investigators. Apixaban versus warfarin in patients with atrial fibrillation. N Engl J Med 2011;365:981–92. 21870978 10.1056/NEJMoa1107039

[68] Cosedis Nielsen J Johannessen A Raatikainen P. Radiofrequency ablation as initial therapy in paroxysmal atrial fibrillation. N Engl J Med 2012;367:1587–95. 23094720 10.1056/NEJMoa1113566

[69] Assi TB Baz E. Current applications of therapeutic phlebotomy. Blood Transfus 2014;12(Suppl 1):s75–83. 24120605 10.2450/2013.0299-12 PMC3934278

[70] Kontoghiorghes GJ Spyrou A Kolnagou A. Iron chelation therapy in hereditary hemochromatosis and thalassemia intermedia: regulatory and non-regulatory mechanisms of increased iron absorption. Hemoglobin 2010;34:251–64. 20524815 10.3109/03630269.2010.486335

[71] Mobarra N Shanaki M Ehteram H. A review on iron chelators in treatment of iron overload syndromes. Int J Hematol Oncol Stem Cell Res 2016;10:239–247. 27928480 PMC5139945

[72] Kowdley KV Brandhagen DJ Gish RG National Hemochromatosis Transplant Registry. Survival after liver transplantation in patients with hepatic iron overload: the national hemochromatosis transplant registry. Gastroenterology 2005;129:494–503. 16083706 10.1016/j.gastro.2005.05.004

[73] Rawla P Sunkara T Muralidharan P. Update in global trends and etiology of hepatocellular carcinoma. Contemp Oncol (Pozn) 2018;22:141–150. 30455585 10.5114/wo.2018.78941 PMC6238087

[74] National Center for Biotechnology Information. Hemochromatosis and Iron Overload Screening (HEIRS) Study. National Center for Biotechnology Information (US); 2003.

[75] Wyse DG Waldo AL DiMarco JP Atrial Fibrillation Follow-up Investigation of Rhythm Management (AFFIRM) Investigators. A comparison of rate control and rhythm control in patients with atrial fibrillation. N Engl J Med 2002;347:1825–33. 12466506 10.1056/NEJMoa021328

[76] Caro JJ. An economic model of stroke in atrial fibrillation: the cost of suboptimal oral anticoagulation. Am J Manag Care 2004;10(14 Suppl):S451–58; discussion S458–61. 15696909

